# Supplementary material for: Constraints on the functioning of Community-based Health Planning and Services facilities: A qualitative study in the Jirapa Municipality, Ghana
Source: PLOS Glob Public Health. 2023 Dec 20;3(12):e0002094. doi: 10.1371/journal.pgph.0002094 (PMC10732441; doi:10.1371/journal.pgph.0002094)
Supplement: Attachment — Submitted filename: Response to Reviewers.docx [file pgph.0002094.s001.docx]

**Authors response to reviewers’ comments**

Reviewer #1:

| **Comment** | **Response** |
| --- | --- |
| 1. Please provide further details about the Jirapa municipality, specifically about the utilization and status of scale up of CHPS - the rationale should justify why this enquiry now and why here? | Thank you for this observation. Further details about the municipality have been provided under the study setting. We have also provided further information on utilization of CHPS in the background of the study to motivate the rationale for the study. Specifically, the following information have been provided  “*In the Jirapa Municipality for instance, childhood vaccination by CHPS for Penta 3, has witnessed a decline from a coverage of 62.3% in 2020 to 62% in 2021 and to 55% in 2022 (Jirapa Municipal Health Directorate, 2023).* |
| 2. What is the composition of CHMC? Do they include community stakeholders? How diverse is it? Is there representation of women and members of all ethnic sects? Further details needed | Typically, CHMC include individuals from a different part of the community; who represent different constituents (youth, women, the aged, etc.). They are basically community stakeholders serving different interest within each CHPS zone. They are relatively diverse and they include women. The Jirapa Municipality is relatively ethnically homogenous so ethnicity is usually not considered when nominating members into the committee.  We have now included the following in the manuscript to respond to the reviewer’s comment  “*CHMC include individuals from communities within the CHPS zone, representing various constituents (youth, women, the aged, etc.) within the catchment area of the CHPS zone*” |
| 3. "This sampling method was used since qualitative researchers often depend on small sample sizes since data saturation was the ultimate goal in this type of research, not the number of participants" - This sampling method was used since qualitative researchers often depend on small sample sizes since data saturation was the ultimate goal in this type of research, not the number of participants. | The statement has been expunged and the appropriate explanation of saturating point clarified as follows:  “*The sample of 51 respondents was deemed adequate because the data collection reached a saturation point, hence data collection ended at the point where the responses given by participants started to become repetitive*” |
| 4. Conditions/criteria for sample selection has been mentioned partially - would you still call it purposive sampling or would you like to define the sampling method using a more refined terminology? Such as criterion sampling or even theoretical sampling? Refer Qualitative Inquiry and Research Design: Choosing Among Five Approaches Book by Cheryl N. Poth and John W. Creswell for further information. | We agree with the reviewer that the respondents were selected based on some criteria which was the unique roles the play in the operations of CHPS and hence their unique capacity to provide the requisite information we were seeking. However, our understanding is that criterion sampling is a type of purposive sampling. . We have now refined our sampling description to include criterion sampling as a form of purposive sampling as a response to the reviewer’s comment as follows  “*A criterion-based purposive sampling technique was used to select the key stakeholders of CHPS as key informants “* |
| 5. Who did the data collection?  Please give a brief description of the background and positionality of the researchers who did the data collection and/or the analysis. The constructivist paradigm mandates that the researchers reflexively engage with the research process and hence, a brief description of one's background, orientation and positionality is needed. | The first author and two trained graduate students collected the data. The two students were selected because of their familiarity with the terrain and culture of the people, their experiences and skills in conducting qualitative studies. We have now clarified this under the methods section as follows:  “*A team of three researchers collected the data. The team included the first author and two trained research assistants. The research assistants were graduate students pursuing courses in public health and possessed extensive qualitative research experience and were familiar with the terrain and the culture of the study area”.* |
| 6. Please provide some more details about the analysis process. for instance, 1. Did you use constant comparison to move from open --> axial --> selective codes. 2. How many people were involved in the coding process? 3. Were the memos discussed within the teams? 4. How were the discordances sorted? 5. Since you committed to grounded theory approach, did you use theoretical sampling also? If yes, what were the initial theoretical categories? 6. You have also committed to a constructivist epistemology, indicating that you are closer to the modified approaches of grounded theory rather than the classical approach. In that case, the researchers' theoretical orientation in co-constructing the knowledge is of significance. Please elaborate on this. | We have now clarified that we adopted a thematic analysis which involved constant comparison of the themes emerging from the sequential coding of the transcripts. We have also clarified that all three authors were involved in the coding and analysis of the data and have explained how consensus was reached on common themes and discordances sorted. In fact, we have extensively revised our description of the data analysis. The claims relating to grounded theory among others have been deleted. |
| 7. The reasons for low community involvement could have been probed further. This would have required a detailed probing of the community level stakeholders and also the system level stakeholders who are experienced richly in the context of Jirapa municipality. | Thank you for this observation. Based on the quotations already presented, we have briefly revised this part of the writeup to put emphasis on lack of refreshment during CHPS activities as the reason that was presented for the low community participation. Unfortunately, we do not have additional data on the reasons for the low community involvement from our study. We have, however, discussed additional plausible reasons for the low community participation drawing from evidence from similar studies within the Ghanaian context in the discussion section. |
| 8. Why did this "lack of understanding of CHPS occur in the community". This would have been an extremely useful enquiry. How did the community develop the "inadequate understanding"? Who transferred the information about CHPS to the community? Were they trained? What modalities did they used for information transfer? Was the process of information transfer adequate in content coverage and appropriate in terms of the mode of delivery? If such critical questions are not asked, the findings would come across as "victim blaming" | Again, we have revised the results section bringing out the perspective of the Municipal coordinator of CHPS on when and how education on the programme is done. We believe that our discussion section has already reflected on these key issues raised by the reviewer. Poor sensitization and communication gaps have already been adduced as reasons for low understanding of CHPS in the discussion section. |
| 9. The failure of authorities to properly drive home the purpose of CHPS to the community could have been highlighted with evidence if the community’s side of the story could also have been presented in a nuanced manner. Isn’t that the ultimate purpose of qualitative research? To paint a holistic and balanced picture of any phenomenon? | We have acknowledged that the study was limited because some community level actors were not included. We have duly included this as a limitation of the study as follows:  “*The study only covered the perspectives of managers and health service providers under the CHPS initiative. The views and perspectives of other stakeholders, especially community level beneficiaries of the CHPS programme are, therefore, missing. Future studies should therefore include the community (service users) perspective on the factors influencing the functioning of CHPS as well.”* |
| 10. The inadequate acknowledgement of religious beliefs and traditional practices in the design of CHPS is an important piece of contextual information which should have informed the implementation of CHPS in this community. Did the system not anticipate this aspect? If not why? | Certainly the CHPS initiative would have anticipated the influence of cultural and religious beliefs system on the functioning of the programme and hence the reason why the programme heavily relies on the participation of various constituents in its activities. Unfortunately, we do not have additional responses from the interviews on this to comment on in the results section. We have, however, already reflected extensively on this issue in the discussion section. |
| 11. Why do these logistic challenges occur? How does the system cope? Have they tried any remedial measures? Have these efforts, if any, produced any results, even if they are localized and of low intensity? | Reviewing the quotations already presented once again, we have included further information about the logistic challenges and local adaptations to those challenges as follows:  “*The interviews revealed that as a local adaption to the logistic challenge, health workers often use their personal motorbikes to carry out CHPS activities while the Municipal Health Directorate commit to servicing and fuelling of such motorbikes. The inability of the CHPS compounds to raise money on their own has made it difficult for such facilities to acquire and maintain the needed tools and logistics required for their effective functioning.”*  We have also discussed this under the discussion section. The logistic challenges occurred as a result of a significant design flaw of the CHPS concept. Funding for CHPS have not been clearly delineated hence there is always a funding gap. CHPS depends on funds from other health institutions within the hierarchy and this causes delays in release of funds. |
| 12. Negative staff attitudes is an important perception that can explain the low utilization by the community. However, in order to build a balanced construction, we need to know what do the system stakeholders think about this perception. How do they make sense of this perception. If they agree that this perception by the community is valid or justifiable, then why do they think this situation exists. A balanced epistemological construction requires us to take the knowledge generated from the one category of stakeholders (system) to another category (community) and vice versa for probing. | The system stakeholders duly acknowledge that these negative staff attitudes existed. The focus of their responses was how these affected the effective functioning of CHPS and the services they delivered. The quotations provided to support this claim also captured the perspective of that of the Municipal CHPS coordinator. All the issues identified in the results have further been elaborated on in the discussion section. As already indicated, the missing perspective of community beneficiaries has been acknowledged as a study limitation. |
| 13. Why does delayed disbursement of NHS claims happen? Is it because of financial constraints at the NHIS or logistic/procedural reasons? The authors can attempt to build potential reasons for this, either from direct interviews with the NHIS level stakeholders or from policy documents or from existing literature. | The delays in disbursement of NHIS funds is caused by multiple factors including but not limited to financial constraints, complicated claims processing procedure, fraud and misappropriation of funds. These have been acknowledged in the discussion section |
| 14. What kind of motivation do the staff actually expect? The descriptions are too trimmed, sanitized and simplistic. Motivation of human resources is an intrinsically complex phenomenon and the authors seem to shy away from delving into the "mess" | Essentially, CHPS staff expect several motivational packages, both financial and non-financial. They expect regular financial packages to motivate them to undertake community outreach as well as performing other duties in remote settings. They also expect nonfinancial support such as the provision of study leave for further studies among others. We have briefly revised the appropriate parts of the manuscript to address this comment |
| 15. Discussion is one of the better written sections of the paper. However, the section can be trimmed and structured better to bring out the "so what" of the findings. Also, it is important that the discussion section brings together all the key findings of the paper and build a coherent narrative/theory for the relatively low utilization of the programme.  I have presented a narrative below to consider:  1. Low community involvement has been identified as one of the key factors which hamper the utlization of the scheme. 2. The relatively low understanding of the scheme by the community and the gaps in the programme design characterized by its poor alignment with the social context, stratification, beliefs and value systems of the community could be the potential reasons for this. 3. The negative attitudes of the system level HR perceived by the community indicates the ineffective nature of knowledge transfer between the system and the community, which could in turn be driven by the financial constraints faced by the system and the poor motivation of the HR.  What are the potential reasons for poor community involvement/participation of any community based health programme? What does international literature say about this? Connect this to each step of the aforementioned narrative. | We thank the reviewer for acknowledging that the discussion section of the paper was well written. We are grateful for the insightful suggestions. We have tried to trim it a bit in response to the comments of the reviewer. We have also reorganized our presentation of the issues to enhance coherence as recommended by the reviewer.  We, however, wish to indicate that it will be improper to indicate as suggested by the reviewer that “…the gaps in the programme design characterized by its poor alignment with the social context, stratification, beliefs and value systems of the community could be the potential reasons for this”. As captured in the introduction of the manuscript, by design the CHPS programme has made provisions for its alignment with the social context, stratification, beliefs and value systems of the communities. However, it is implementation challenges that should be blamed for the challenges reported. As revealed by our study and discussed, poor sensitization of communities partly resulting from inadequate training of staff at the CHPS level are responsible for the low understanding of the concept. |
| 16. In the discussion section, it is critical to connect the negative staff attitudes to their poor motivation and acknowledge the systemic causes for this. Otherwise it looks like we are unjustly labelling the health workers working in a public sector which is marred by financial constraints. The construction should be made more balanced to avoid "blaming" | Thank you for this observation. We have amended that section to reflect this suggestion |
| 17. Findings cannot be generalized is NOT a limitation of qualitative research. Qualitative research is built on constructive/transformational paradigms which are based on the ontological and epistemological assumptions that reality is multiple and is socially constructed. Here, our objective is to build balanced and holistic constructions of "how" and "why" social phenomena occur. Please revisit the statement. | We have expunged the sentence and indicated the main limitation of the study. |
| 18. Overall copyediting and English correction is recommended | We apologize for the errors and have thoroughly edited and revised the manuscript to address these issues. |

Reviewer #2:

| **Comment** | **Response** |
| --- | --- |
| 1) The manuscript requires copyediting. Sentence formation need to be changed in certain places which are marked with comments in the uploaded reviewer document | We are grateful to the reviewer for pointing out those errors for us to address. We have thoroughly edited and revised the manuscript in order to address these issues |
| 2) In methodology portion few things need to be elaborately described particular places are marked in the file. | We have effected all suggested changes. Additional information have also been provided as requested |
| 3) Stating generalizability as a limitation is not acceptable as qualitative study is not meant for generalizability. | This statement has been duly expunged and the main limitation of the study indicated. |
| 4) All the comments in the file may be addressed for the fine tuning of the manuscript. | We are grateful for the very insightful comments and suggestions. We have effected all the necessary changes as suggested. |
|  |  |

.
