# Supplementary material for: Constraints on the functioning of Community-based Health Planning and Services facilities: A qualitative study in the Jirapa Municipality, Ghana
Source: PLOS Glob Public Health. 2023 Dec 20;3(12):e0002094. doi: 10.1371/journal.pgph.0002094 (PMC10732441; doi:10.1371/journal.pgph.0002094)
Supplement: Attachment — Submitted filename: Authors response to comments.docx [file pgph.0002094.s003.docx]

PGPH-D-23-00302R1
Constraints on the functioning of Community-based Health Planning and Services facilities: A qualitative study in the Jirapa Municipality, Ghana
PLOS Global Public Health

**Response to reviewer comments**

**Reviewers’ comments**

Reviewer #1: • There are no unjustified claims about the methodology employed
• Sufficient details have been provided about the methods used
• Conclusions are balanced and justified
• Benefitted from an overall copyediting
• Statements which undermined the paradigm have been removed
• I recommend the paper for publication

Reviewer #2: (No Response)

**Authors response:** We are very grateful to the two reviewers for the positive comments about our manuscript and for recommending it for publication. We have observed that the second reviewer did not provide any comments for us to address. Also, all the comments by the first reviewer are positive confirming that all his/her initial comments were adequately addressed. No additional comments were provided by the editors or reviewers for the authors to address after the second round of the review. We, therefore, conclude that no changes are needed in response to reviewers’ comments.

We have also thoroughly proof-read the manuscript again and all additional errors detected have all been corrected.
