# Supplementary material for: Constraints on the functioning of Community-based Health Planning and Services facilities: A qualitative study in the Jirapa Municipality, Ghana
Source: PLOS Glob Public Health. 2023 Dec 20;3(12):e0002094. doi: 10.1371/journal.pgph.0002094 (PMC10732441; doi:10.1371/journal.pgph.0002094)
Supplement: Attachment — Submitted filename: Report to PLOS.docx [file pgph.0002094.s005.docx]

Dear Editor,

Thanks for the opportunity to review this manuscript. My recommendations are below.

1) The title should meet the SMART requirements.

2) Researchers should write down the inclusion and exclusion criteria in more detail.

3) The result part is just putting the idea; what about the idea saturation issue? You have to express

4) In the recommendation part, it is general it should be specific to concerned body.

5) References should be up to date, old references example reference number 6.

Best regards
